# A review of the flortaucipir literature for positron emission tomography imaging of tau neurofibrillary tangles

**DOI:** 10.1093/braincomms/fcad305

**Published:** 2023-11-16

**Authors:** Samantha C Burnham, Leonardo Iaccarino, Michael J Pontecorvo, Adam S Fleisher, Ming Lu, Emily C Collins, Michael D Devous

**Affiliations:** Avid, Eli Lilly and Company, Philadelphia, PA 19104, USA; Avid, Eli Lilly and Company, Philadelphia, PA 19104, USA; Avid, Eli Lilly and Company, Philadelphia, PA 19104, USA; Avid, Eli Lilly and Company, Philadelphia, PA 19104, USA; Avid, Eli Lilly and Company, Philadelphia, PA 19104, USA; Avid, Eli Lilly and Company, Philadelphia, PA 19104, USA; Avid, Eli Lilly and Company, Philadelphia, PA 19104, USA

**Keywords:** Alzheimer’s disease, neurofibrillary tangles, flortaucipir, neuroimaging, tau

## Abstract

Alzheimer’s disease is defined by the presence of β-amyloid plaques and neurofibrillary tau tangles potentially preceding clinical symptoms by many years. Previously only detectable post-mortem, these pathological hallmarks are now identifiable using biomarkers, permitting an *in vivo* definitive diagnosis of Alzheimer’s disease. ^18^F-flortaucipir (previously known as ^18^F-T807; ^18^F-AV-1451) was the first tau positron emission tomography tracer to be introduced and is the only Food and Drug Administration-approved tau positron emission tomography tracer (Tauvid™). It has been widely adopted and validated in a number of independent research and clinical settings. In this review, we present an overview of the published literature on flortaucipir for positron emission tomography imaging of neurofibrillary tau tangles. We considered all accessible peer-reviewed literature pertaining to flortaucipir through 30 April 2022. We found 474 relevant peer-reviewed publications, which were organized into the following categories based on their primary focus: typical Alzheimer’s disease, mild cognitive impairment and pre-symptomatic populations; atypical Alzheimer’s disease; non-Alzheimer’s disease neurodegenerative conditions; head-to-head comparisons with other Tau positron emission tomography tracers; and technical considerations. The available flortaucipir literature provides substantial evidence for the use of this positron emission tomography tracer in assessing neurofibrillary tau tangles in Alzheimer’s disease and limited support for its use in other neurodegenerative disorders. Visual interpretation and quantitation approaches, although heterogeneous, mostly converge and demonstrate the high diagnostic and prognostic value of flortaucipir in Alzheimer’s disease.

## Teaser

The available literature provides substantial evidence for the use of flortaucipir in assessing neurofibrillary tangle pathology in Alzheimer’s disease and limited support for its use in other neurodegenerative disorders. Visual interpretation as well as quantitation approaches demonstrates the high diagnostic and prognostic value of flortaucipir in Alzheimer’s disease.

## Graphical Abstract

**Graphical Abstract:**
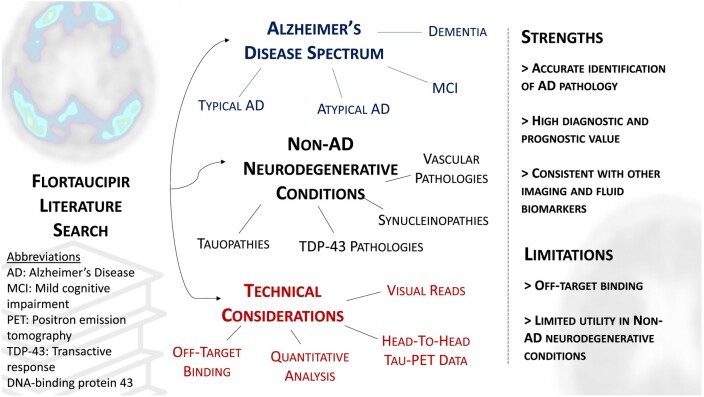

## Introduction

Alzheimer’s disease is a clinicopathological entity^1,2^ characterized by progressive memory loss, cognitive dysfunction and the inability to perform activities of daily living. Alzheimer’s disease is the major cause of dementia and the seventh leading cause of death in the USA, with prevalence increasing as the population ages.^1,3-6^ The defining hallmark features of Alzheimer’s disease are β-amyloid (Aβ) plaques and tau neurofibrillary tangles (NFTs).^1,3^ Previously only detectable post-mortem, positron emission tomography (PET) biomarkers^1,7,8^ now permit a pathological diagnosis before death. These biomarkers aid in diagnosis, staging of disease, evaluating disease progression and assessing the efficacy of potential therapeutics. Flortaucipir (previously ^18^F-T807; ^18^F-AV-1451) is currently approved by the U.S. Food and Drug Administration (FDA) as Tauvid™: ‘a radioactive diagnostic agent indicated for PET imaging of the brain to estimate the density and distribution of aggregated tau neurofibrillary tangles (NFTs) in adult patients with cognitive impairment who are being evaluated for Alzheimer’s disease.’^9^ It was the first tau PET tracer to be introduced and has been widely adopted and validated in several research and clinical settings. Here, we present an overview of the published literature on flortaucipir PET imaging of NFTs. We considered all accessible peer-reviewed literature from a PubMed search of the term ‘(Flortaucipir) OR (Tauvid) OR (AV1451) OR (“AV 1451”) OR (“AV-1451”) OR (T807) OR (T-807) OR (“T 807”)’ covering all available dates until 30 April 2022; 605 papers were identified of which 462 were included and 143 were excluded as they were not original research articles or relevant to flortaucipir. An additional 12 papers that did not meet search criteria were included as they were considered to be relevant to this review.

## Flortaucipir in typical Alzheimer’s disease, MCI, and pre-symptomatic populations

Much of the published literature^10-28^ indicates that the binding of flortaucipir to tau NFTs parallels NFT accumulation described as Braak stages,^10,29-31^ especially for Aβ-positive subjects,^30,32^ and correlates with clinical symptoms and measures of neurodegeneration.^33^ Braak Stages I and II are used when NFT involvement is predominant in transentorhinal, entorhinal and hippocampal regions, Stages III and IV when limbic regions, medial and inferior temporal lobes are involved and Stages V and VI when NFTs may be found widespread in the neocortex.^29^ Distinct Braak stage-specific patterns of flortaucipir binding were reported in a proportion like that seen at autopsy in patients with Alzheimer’s disease.^34^ Regional flortaucipir distribution in most cases paralleled the expected (Braak-like) spatial progression and in turn was significantly associated with amyloid status, diagnostic category and measures of global cognition.^35^

It should be noted that throughout the review, references to clinical syndromes (mild cognitive impairment [MCI], dementia, etc.) may or may not be supported by evidence of the underlying aetiology (Alzheimer’s pathology, etc.). In general, the terminology reflects that utilized in the specific literature to ensure clarity in the reporting of those findings.

### Flortaucipir positron emission tomography and pathology at autopsy

Flortaucipir penetrates the blood–brain barrier to bind NFTs, allowing *in vivo* visualization of the density and distribution of neocortical NFT accumulation. Its correlation with NFT pathology at autopsy has been demonstrated by several studies.

A study^36^ evaluating flortaucipir and autopsy findings in 64 patients had a short mean difference of 2.6 months between flortaucipir scan and autopsy. The standard of truth was tau pathology consistent with Braak Stages V and VI or high Alzheimer’s disease neuropathological change (ADNC) consistent with Braak Stages V and VI NFT pathology and extensive Aβ plaque pathology. The ADNC score can be ‘none, low, intermediate and high’,^37^ and such determination is based on three different pathology scores capturing (i) amyloid plaques with the Thal phases method^38^; (ii) NFT stage with the Braak method^29^ and (iii) neuritic Aβ score with the Consortium to Establish a Registry for Alzheimer's Disease (CERAD) method.^39^ A high ADNC score corresponds to advanced Thal (Thal Phase 4/5) and Braak stages (Braak V or VI), with at least moderate CERAD score.^37^ Majority read of the visual evaluation by five raters found that flortaucipir had a sensitivity of 92.3% and specificity of 80.0% for identifying Braak Stages V and VI NFT pathology as well as a sensitivity of 94.7% and specificity of 80.8% for identifying high ADNC.^36^ The study was replicated with a separate five readers and an additional 18 patients (*n* = 82) with similar findings.

A second study^40^ evaluated autopsy findings and quantitative flortaucipir (*n* = 26) using the standard uptake value of flortaucipir in a meta-region of interest (ROI) consisting of amygdala, entorhinal cortex, fusiform, parahippocampal as well as inferior temporal and middle temporal gyri and were normalized to the cerebellar crus to obtain standardized uptake value ratios (SUVrs). All participants with NFT pathology of Braak Stage IV or greater had an elevated (abnormal) flortaucipir SUVr, which identified Alzheimer’s disease spectrum pathology with a sensitivity of 87% and specificity of 82%. This study only had two participants at Braak Stage IV, which were close to the internally derived threshold, and in a follow-up study with 143 subjects, it was stated that elevated flortaucipir SUVr was associated with Braak V/VI.^41^ Similarly, high correspondence was found between *in vivo* flortaucipir retention and neuropathology in Alzheimer’s disease patients (*n* = 8) with flortaucipir SUVr quantitation reliably detecting advanced Alzheimer’s disease pathology (Braak VI).^42^ However, participants with earlier Braak stages (Braak I–IV) did not show elevated tracer uptake compared with young, tau-negative controls.

In addition to autopsy measures of NFT stage and Aβ, flortaucipir also correlates with immunohistology measures of tau. These important results support the finding that flortaucipir maps to the density as well as the distribution of NFTs in the brain. Further, quantitation of cortical regional flortaucipir binding was found to be significantly correlated with quantitative P-tau immunohistochemistry,^40,43,44^ In this case study, flortaucipir also significantly (*P* < 0.001) correlated with other post-mortem findings, including density of tau-positive neurites, intrasomal tau tangles and total tau burden. Another study (*n* = 22)^45^ provided evidence that flortaucipir bound in regions precisely mirroring the pattern of P-tau immunohistology staining across different Braak stages.

### Flortaucipir and amyloid positron emission tomography

Aβ plaques, another Alzheimer’s disease hallmark feature, can be assessed *in vivo* using FDA and European Medicines Agency-approved PET imaging tracers (^18^F-florbetapir, ^18^F-florbetaben and ^18^F-flutemetamol) and research-only tracers [^18^F-flutafuranol (NAV4694) and ^11^C-Pittsburgh Compound B]. Generally, elevated flortaucipir signal (global as well as regional) is reported to occur in the presence of elevated global amyloid-PET signal.^10,11,13,14,18,19,23,46-52^

A recent study specifically investigated whether elevated global flortaucipir signal would be an accurate predictor of amyloid-PET positive status, finding 98% accuracy.^53^ Several other studies have provided data in support of these observations, overall showing that regardless of the method adopted (e.g. the threshold or quantitation approach used), elevated neocortical flortaucipir signal was rarely observed in patients without concomitant elevated neocortical amyloid-PET signal. In clinically heterogeneous cohorts, 81–100% of individuals with elevated flortaucipir signal also exhibited elevated amyloid-PET signal.^12,14,35,54-60^ Even in a predominantly MCI cohort, 72% of those with elevated flortaucipir signal limited to the medial temporal region (likely an earlier measure of tau positivity than neocortical signal) also had elevated neocortical amyloid-PET signal.^61^ These findings imply that elevated flortaucipir signal may support the identification of both NFT and Aβ plaque pathology, consistent with the autopsy results (above). That is, elevated flortaucipir indicated both Braak V/VI NFT levels and high ADNC (Braak V/VI plus elevated amyloid). This observation is consistent with neuropathology data from autopsy studies, showing that an advanced Braak V/VI NFT stage is in most cases associated with widespread and dense amyloid plaque accumulation.^62^

These findings also align with suggestions that global tau, specifically tau beyond the medial temporal lobe, is associated with, and may be dependent on, Aβ accumulation.^55,63,64^ Some investigators postulate that increased Aβ accumulation might affect subsequent spread of tau via intrinsic neuronal networks.^13,23,63^ However, early stage flortaucipir binding in regions such as medial temporal lobe has been observed in the absence of elevated global Aβ, in a manner similar to primary age-related tauopathy (PART).^61,65-67^ Thus, a sequential cascade of events is hypothesized where local, medial temporal tau precedes global Aβ pathology, which, in turn, precedes global tau pathology. This cascade is supported by reports of the rate of Aβ accumulation^68^ and baseline Aβ^69-72^ predicting flortaucipir accumulation. In addition, antecedent elevations in Aβ, primarily in inferior temporal and parietal regions, were observed ∼5 years prior to elevated flortaucipir, primarily in inferior temporal and parietal regions^18,73,74^

Although elevated flortaucipir signal was consistently shown to be associated with an elevated amyloid-PET scan, the reverse was not true—an elevated amyloid-PET scan was not always associated with an elevated flortaucipir scan. Similarly, a negative flortaucipir PET scan was not always associated with a negative amyloid-PET scan. It should be noted, however, that one study suggested high levels of Aβ binding (Centiloid >50CL) at baseline were indicative (80% sensitivity and specificity) of an elevated flortaucipir scan 5 years later.^74^ Even in non-Alzheimer’s disease dementia and cognitively unimpaired (CU) participants, elevated amyloid-PET SUVr was shown to be the strongest predictor of subsequent elevated flortaucipir signal.^75^

Studies have found that flortaucipir SUVr was significantly higher for Aβ+ MCI and Alzheimer’s disease relative to the respective Aβ− diagnostic groups.^55^ Flortaucipir SUVr was higher for the Aβ+ versus Aβ− groups irrespective of neurodegeneration status^76^ and among subjects without elevated tau (tau−).^69^ However, in the neurodegeneration negative group, flortaucipir deposition was limited to the medial and lateral temporal regions.^76^ One study showed that, in comparison with younger CU subjects, older Aβ− CU subjects showed increased flortaucipir signal restricted to the medial temporal lobe, but no difference in other neocortical regions (e.g. suggestive of PART). There were no differences in flortaucipir uptake among older impaired versus unimpaired Aβ− subjects.^55^

While current thinking generally aligns with amyloid and tau CSF markers becoming abnormal earlier in the disease cascade than amyloid and tau PET markers,^77-79^ some conflicting reports suggest similar or higher prevalence of PET+/CSF− in comparison with PET−/CSF+.^56,80-82^ Further, there are conflicting reports on the distribution and density of flortaucipir binding with different CSF and PET profiles.^83,84^

In a head-to-head cross-over study comparing the clinical utility of both flortaucipir and amyloid-PET evaluations,^85^ both had a significant and similar impact on diagnosis and diagnostic confidence, and adding one to the other further improved diagnostic confidence. This agreed with findings that combining amyloid and tau PET may help with differential diagnosis and prognosis.^86^

A discussion on the association of flortaucipir and non-amyloid PET tracers is provided in Supplementary material.

### Flortaucipir and CSF

CSF and plasma markers (discussed below) provide estimates of soluble circulating protein levels, such as amyloid and tau proteins.^87^ For amyloid, common markers include Aβ42 and the Aβ42/Aβ40 ratio.^2,88,89^ The most common and currently validated tau markers are total tau protein (t-tau) and tau phosphorylated at threonine 181 (P-tau181), threonine 217 (P-tau217) or threonine 231 (P-tau231).^2,90^ CSF t-tau assays measure the amount of tau protein in CSF, regardless of phosphorylation, and are generally considered the markers of neurodegeneration. Phosphorylated tau is a measure of dysregulated tau metabolism and processing and is representative of disease state (intensity of the disease process). It is hypothesized that abnormal phosphorylated tau contributes to NFT accumulation.^7^

Alzheimer’s disease/MCI individuals have abnormally low levels of CSF Aβ42, lower Aβ42/Aβ40 CSF ratios and higher levels of CSF P-tau, t-tau and correspondingly high flortaucipir PET signal compared with CU.^3,54,91-95^ Several groups have shown that flortaucipir global SUVr significantly correlates with CSF t-tau (*r* = 0.37–0.80), P-tau181 (*r* = 0.29–0.75), P-tau217 (*r* = 0.78) and P-tau181/Aβ42 (*r* = 0.73); however mixed findings are reported for CSF Aβ42 (*r* = −0.49 to 0.02).^10,24,25,49,96-102^

Studies indicated that relative to flortaucipir PET, CSF P-tau181 had concordance of 71–87%^25,103-106^ and CSF P-tau217 had concordance of 74–80%.^103,106^ In head-to-head studies, both correlation^78^ and concordance^106^ of flortaucipir are reported to be higher with P-tau217 compared with P-tau181. P-tau181/Aβ40 ratio has improved performance over P-tau181 alone.^107^ Evaluations of discordance suggest that tau CSF+/PET− is more common than the reverse,^104,108^ and discordance is more prevalent in earlier disease stages,^105^ supporting the sequential cascade hypothesis that tau phosphorylation precedes NFT deposition.^109,110^ Flortaucipir, CSF P-tau181 and CSF t-tau all had high accuracy in identifying dementia due to Alzheimer’s disease or MCI due to Alzheimer’s disease (area under the curve [AUC] 0.84–1.00).^24,25,100^ Flortaucipir is, however, reportedly a better predictor of neurodegeneration, cognitive performance and cognitive decline in comparison with CSF t-tau and P-tau181 or 217 measures.^25,100,111,112^ This aligns with tau-PET being an estimate of the overall burden of aggregated tau pathology and being downstream of peripheral phosphorylated tau changes in the sequential cascade of biomarkers.^10,113^

CSF t-tau and P-tau181 are reported to correlate significantly with flortaucipir PET signal in entorhinal, limbic, parahippocampal, inferior temporal, temporoparietal, medial prefrontal and medial temporal regions (partial *r* = 0.53–0.73).^49,56,96,100,102,103,111,114^ Lower CSF Aβ42 predicted higher flortaucipir binding in temporal lobe but not in limbic regions,^100^ perhaps consistent with the hypothesis that tau accumulation begins in the mesial temporal lobe but the development of tau beyond the mesial (limbic) temporal lobe is associated with, and may be dependent on, amyloid accumulation.^55,62,63^ The variations in correlation and concordance between flortaucipir and CSF measures are at least partially reflective of different populations, assays, thresholds and imaging methods. For example, the relationships of CSF t-tau or P-tau181 are specifically stated to differ based on the stage of disease and region of flortaucipir uptake.^10,114^ The distribution and density of flortaucipir signal is also reported to be different between those who are positive for Aβ as assessed by either PET or CSF.^84^

CSF markers other than Aβ and tau have also been compared with flortaucipir. These CSF markers include glucose, which was associated with lower flortaucipir binding in Braak Stage I/II in women and *APOE* ɛ4 carriers,^115^ apoB levels, which correlated with flortaucipir binding in the entorhinal, parahippocampal and fusiform regions,^116^ tumor necrosis factor alpha (TNF-α) and interlleukin 8 (IL-8), which were negatively correlated with flortaucipir in the medial temporal lobe,^117^ and neurofilament light chain (NfL) which was correlated with higher flortaucipir binding.^118^ Synaptosomal-associated protein, 25 kDa, growth-associated protein 43 (GAP-43) and neurogranin were not shown to correlate with flortaucipir.^118^

### Flortaucipir and blood biomarkers

Like CSF, the primary blood markers of interest in Alzheimer’s disease pathology are amyloid (Aβ42, Aβ42/Aβ40 ratio) and tau (P-tau, t-tau). There is an emerging literature showing relationships between these plasma markers and tau-PET imaging. Plasma markers for Alzheimer’s disease pathology, however, are relatively new, and their performance is likely to be impacted by the given marker (e.g. Aβ42/Aβ40, P-tau181, P-tau217, P-tau231), as well as by which assay/platform the analyses are conducted on (e.g. mass spectrometry versus immunoassay)^119^; however, overall the findings support the hypothesis that blood (and CSF) markers become abnormal prior to PET biomarkers. Lower plasma Aβ42/Aβ40 ratios are associated with increased flortaucipir binding in Alzheimer’s disease-related regions,^120^ and plasma Aβ42/Aβ40, P-tau181 and P-tau217, but not NfL, had robust discrimination performance reported for identifying elevated flortaucipir binding.^121,122^ Plasma P-tau, t-tau, P-tau/Aβ42 and t-tau/Aβ1–42 ratios had significant correlations with flortaucipir binding in both cross-sectional and longitudinal analyses.^99,101,103,121^ Additionally, plasma P-tau181, as well as P-tau231, correlates with flortaucipir SUVr in medial, temporal, parietal and inferior temporal regions, and plasma P-tau231 also correlates with flortaucipir SUVr in the lateral parietal and occipital cortices.^123^ It was also reported that plasma P-tau181 was correlated with cortical flortaucipir SUVr as well as frontal, precuneus, temporoparietal and posterior cingulate SUVrs.^121^

The relationship between plasma P-tau181 and flortaucipir was stronger with increasing clinical disease severity.^101^ One study^109^ reports that plasma P-tau181 becomes abnormal simultaneously with CSF P-tau181, but before flortaucipir, and this finding was further supported by a second study.^124^ Similar to the CSF findings, reported above, this suggests that plasma P-tau markers become abnormal prior to flortaucipir signal and aligns with findings that plasma P-tau correlates with both amyloid-PET and tau-PET,^121^ further supporting the sequential cascade of biomarker abnormality hypothesized in Alzheimer’s disease progression.^113^

Additional studies looking at exploratory markers found that plasma N-terminal tau (NT1) was associated with flortaucipir binding.^125^ In contrast, evaluations of flortaucipir and serum uric acid,^126^serum zinc,^127^ haemoglobin^128^ or homocysteine^129^ found no associations.

Blood biomarkers for Alzheimer’s identification represent a fast-moving landscape, and much has changed since the cut-off date for this literature review. It should be noted that new horizon biomarkers, such as MTBR species, have been proposed that may have a higher concordance with tau-PET.^130^

### Flortaucipir and cognition

Several cross-sectional studies report that increasing global flortaucipir is associated with worse performance on global cognition,^11,16,19,23,46,55,131^ more severe functional impairment^21,131,132^ as well as more severe memory impairment.^16,23,55,131,133^ Similar observations have been described in CU subjects, where higher levels of flortaucipir were associated with worse performance on memory^52,134,135^ and digital cognition tests.^136^

Additionally, a greater extent of binding was associated cross-sectionally with worse performance on global cognition.^11,59,95^ Others have reported on associations between regional flortaucipir binding and specific cognitive domains or tasks. Elevated flortaucipir binding in the medial temporal lobe, especially the entorhinal cortex, is associated with poorer performance on measures of global cognition^137-139^ and memory.^10,14,17,46,48,61,138,140,141^ Visuospatial impairment has been observed to associate with elevated flortaucipir in the occipital lobe and right temporal regions,^14^ whereas language impairment has been associated with elevated flortaucipir in parts of the language network (including lateral parietal and inferior frontal regions), as well as with occipital and temporal areas,^14,140^ and executive/attentional deficits were found to be associated with elevated flortaucipir in lateral temporal, parietal and frontal regions.^142^

Elevated flortaucipir binding is also a robust predictor of subsequent global cognitive decline,^58,95,106,143-146^ with increasing flortaucipir binding in the entorhinal cortex, anterior-temporal and posterior medial regions having a reported association with memory decline as well as clinical progression from pre-symptomatic Alzheimer’s disease to MCI/Alzheimer’s disease.^147-150^ In CU populations, increased temporal flortaucipir binding is associated with greater memory decline,^147,151,152^ but not with change in executive function.^153^ Of note, such association between elevated flortaucipir binding and longitudinal memory decline in CU participants was only observed within amyloid-PET positive subjects.^153^ Similarly, higher flortaucipir binding was associated with decreased learning (practice effects)^154,155^ and increased reporting of subjective cognitive deficit in CU subjects.^156^ Even within participants with subjective memory complaints, increased medial temporal lobe flortaucipir correlated with greater subjective cognitive decline as well as Aβ burden.^157,158^

Flortaucipir, amyloid-PET, CSF biomarkers and atrophy are all associated with cognitive performance and decline; however, flortaucipir has a stronger relationship with cognition than the other biomarkers,^58,59,94,159^ with one contrasting report where amyloid-PET was more strongly associated with the Montreal Cognitive Assessment in 20 MCI participants.^49^ Further, the relationship of regional flortaucipir with cognition is also reportedly modified by amyloid-PET, age and comorbid pathologies.^61,153,160-162^ One report found significant negative correlations between flortaucipir uptake in early tau accumulation regions (e.g. medial temporal areas) and cognition, in patients without dementia, across memory, executive and language domains. These associations were particularly significant in A+/T+ subjects who were both amyloid and tau-PET positive, but remained significant, although to a lower magnitude, even when restricting the analyses to amyloid-PET–negative, tau-PET–positive subjects.^61^

Increased flortaucipir is associated with increased anosognosia, loneliness, apathy, neuroticism, depression and anxiety as well as loss of conscientiousness, openness, worse road driving skill, decreased financial capacity and reduced ability to cope with stress.^163-174^ Braak Stage I/II ROI flortaucipir SUVr has an accuracy of 78% in identifying participants with affective symptoms.^167^

These studies are all consistent with the neuropathological literature that the spatial location and density of tau may indicate the characteristics of the cognitive deficits, as well as the degree of neurodegeneration and synaptic dysfunction.

### Flortaucipir and structural MRI

The relationship between flortaucipir and neurodegeneration as evidenced by changes in MRI has been examined extensively.^14,17,21,27,46,175-185^ These studies suggest a relationship between flortaucipir and neurodegeneration and also that each contribute independently to measures of cognitive impairment.^146^ Specifically, flortaucipir is more sensitive than volumetric measures at detecting early cognitive changes in pre-clinical Alzheimer’s disease, but both measures correlate strongly with cognition at the clinical stages of Alzheimer’s disease.^58^

Although negative associations between flortaucipir binding and cortical thickness are reported,^49,186,187^ the relationship with atrophy is more complex with regional differences reported.^49,167,188,189^ However, baseline flortaucipir measures were shown to predict longitudinal changes in atrophy,^69,183,184,188,190^ with the distribution of flortaucipir binding being predictive of the topology of future atrophy.^191,192^

Correlations between flortaucipir and glucose hypometabolism are generally stronger than between atrophy and hypometabolism.^181,184,193^ Further, correlations among flortaucipir uptake, hypometabolism and cortical thickness are strongest in participants with greater cortical tau burden.^181,184,193^

Finally, CU individuals with a high degree of enlarged centrum semiovale perivascular spaces had an odds ratio of 2.24 for elevated flortaucipir signal in comparison with those without a high degree of enlarged centrum semiovale perivascular spaces.^194^

### Flortaucipir and other imaging modalities

Functional connectivity MRI or diffusion tensor imaging studies^176,181,195-207^ suggest that more densely interconnected brain regions have higher flortaucipir binding in Alzheimer’s disease dementia and are associated with reduced functional connectivity. Entorhinal flortaucipir binding is correlated with hippocampal activation during visual item memory encoding.^208^ Higher functional connectivity was associated with higher covariance in flortaucipir SUVr, regardless of the presence of cognitive impairment (MCI or dementia), amyloid deposition or small vessel disease (SVD).^200^ Of note, this study demonstrated that brain regional functional connectivity patterns were predictive of regional tau-PET signal patterns, potentially supporting the notion that functional connectivity facilitates the seeding and propagation of tau.^200^ Although in one study of individuals with elevated Aβ, connectivity was lower in those with elevated flortaucipir binding.^201^ Another study^209^ reported reductions in the hippocampal connectome correlated with flortaucipir binding in the hippocampus in both MCI and Alzheimer’s disease dementia participants, whereas another study^210^ reported that flortaucipir binding was not associated with functional MRI response amplitude.

It was found that a dual-phase relationship exists in amyloid-positive individuals with increased connectivity with low neocortical tau and decreased connectivity with elevated flortaucipir levels, suggesting hyperconnectivity followed by hypoconnectivity phases during pre-clinical Alzheimer’s disease.^201^ Flortaucipir binding did not impact the association between default mode network and cortical thinning in participants with abnormal amyloid-PET.^211^

The relationship between Alzheimer’s disease pathology and measures of vascular comorbidity is still not well understood, although a number of MRI studies have started to investigate such relationships. One study^204^ observed that flortaucipir, but not amyloid-PET, was associated with white matter integrity loss. Another study^212^ reports that regions with white matter hyperintensity also exhibited higher flortaucipir binding. Cerebral microhaemorrhages were associated with global flortaucipir binding, and more extensive microhaemorrhages predicted future parietal flortaucipir binding.^213^ Increased flortaucipir binding was associated with lower cerebral blood flow in the entorhinal cortex measured through arterial spin-labelled MRI,^214^ as well as in the lateral temporal and parietal regions.^142^ Increased flortaucipir binding was also associated with lower global blood flow measured by pseudocontinuous arterial spin-labelled MRI.^215^ Additionally, quantitative susceptibility mapping was significantly associated with flortaucipir uptake in the basal ganglia,^216^ and increased tissue sodium levels, measured through 7T sodium MRI, were associated with higher flortaucipir binding.^217^

Overall, these studies seem to support the notion that vascular changes are associated with tau pathology, potentially at difference with amyloid pathology, as also demonstrated by autopsy data.^218^ Future studies systematically investigating the relationship between cerebrovascular disease and Alzheimer’s disease pathology^219^ are warranted to potentially guide the design of future clinical trials and/or interventions.

Few studies have investigated the relationship between flortaucipir-PET and magnetoencephalography signals in Alzheimer’s disease patients.^220,221^ These studies demonstrated how flortaucipir-PET signal elevation co-localizes with and modulates different alterations in neural synchrony,^220^as well as how flortaucipir-PET signal elevation associates with abnormal neural oscillations.^221^

### Flortaucipir with clinical treatment trials

Flortaucipir has been used as both an inclusion criterion and secondary end-point in clinical treatment trials. Flortaucipir was used to identify a low-medium tau population for inclusion in the anti-amyloid therapy donanemab Phase 2 trial, which returned positive results.^222^ Further, it was used to stratify patients in the donanemab Phase 3 trial, which also returned positive results.^223^ Data from the Phase 3 donanemab trial suggested that patients with a lower disease burden as measured by tau-PET received a greater benefit from the treatment. Donanemab is an antibody directed at the pyroglutamate modification of the third amino acid of the amyloid beta epitope that is present only in brain amyloid plaques. Treatment with donanemab was shown to reduce cerebral amyloid plaques and slow the rate of disease progression.

The increase from baseline in regional flortaucipir was significantly lower in donanemab-treated patients relative to the placebo-treated patients in the Phase 2 donanemab study; however, global flortaucipir was reported to be unchanged in both the Phase 2 and Phase 3 studies.^222-224^ No impact on flortaucipir binding was reported for treatment with LY3202626 (a β-secretase 1 inhibitor),^225^ rasagiline^226^ or lanabecestat.^224,227^ Mixed findings were reported for cholinesterase inhibitors with one study showing slower longitudinal increases of flortaucipir binding with treatment^228^ and a second suggesting that donepezil treatment was reported to be associated with higher flortaucipir binding in MCI patients.^229^ Increased change from baseline in flortaucipir binding was found with aducanumab treatment in a single case study.^230^ These studies highlight the ongoing use and importance of flortaucipir to monitor and elucidate the consequences of putative therapeutic treatments for Alzheimer’s disease^229^; further, it is anticipated that flortaucipir may be even more useful as an outcome marker for tau targeting therapies.

### Flortaucipir and other measures

*APOE* ɛ4 carriers generally have greater flortaucipir binding and/or accumulation in comparison with non-carriers,^14,231-234^ and this is reported to follow a medial temporal lobe predominant pattern.^235^ In addition, *APOE* ɛ4 carriers with elevated Aβ had increased flortaucipir binding; with homozygotes, additionally having a more widespread pattern of binding.^50^ The relationship between *APOE* ɛ4 carriage and flortaucipir is reported to be both independent^74,231^ and dependent on interactions with sex,^236-239^ Aβ^50,232^ and/or age^190^ in conflicting reports. Women *APOE* ɛ4 carriers were reported to have higher flortaucipir binding in comparison with women non-carriers^236^ and men,^236,239^ and men homozygotes were reported to have higher flortaucipir binding than men heterozygotes.^239^ A three-way interaction among age, Aβ, and *APOE* ɛ4 carriage was also reported^240^ where younger (66–70-year-old) *APOE* ɛ4 carriers with elevated Aβ had significantly higher flortaucipir binding than non-carriers, but that older (74–78 year old) *APOE* ɛ4 carriers with elevated Aβ did not have elevated binding in comparison with non-carriers.

There is a large body of evidence in support of a complex relationship between age and flortaucipir PET signal. This complexity may be due to the entanglement of the role of tau in the natural history of Alzheimer’s disease pathogenesis and in the normal ageing process. For cognitively impaired patients in the Alzheimer’s disease continuum, i.e. with a positive amyloid status, younger age has been consistently shown to associate with higher neocortical flortaucipir PET signal, especially in lateral temporal, inferior and superior parietal and frontal regions.^14,55,98,140,190,235,241-248^ Our hypothesis is that patients with a younger age of onset may present with a higher burden of NFT pathology in comparison with those with a later disease onset. Contrastingly, in CU individuals, increasing age has been predominantly observed to positively correlate with focal increases in medial temporal flortaucipir PET signal, with some evidence for focal neocortical associations in amyloid-positive CU subjects.^12,14,23,102,152,249^ Flortaucipir binding is greater in women compared with men,^72,151,233,250-254^ although flortaucipir binding also has a strong relationship with the interaction between sex and *APOE* ɛ4 genotype.^255^ No differences in flortaucipir by race (African American and white) were reported.^256^ In patients of equal cognitive impairment, education was correlated with flortaucipir binding,^257-259^ suggesting that with higher levels of education more tau pathology is necessary to induce cognitive deficit, consistent with the cognitive reserve hypothesis.^259,260^

There have been studies examining associations between flortaucipir binding with obstructive sleep apnoea,^261,262^ higher cardiovascular risk metrics,^263^ lower self-reported sleep,^264^ lower self-reported physical activity,^265^ birth season,^266^ visual contrast sensitivity,^267^ more fragmented networks measured with electro-magnetoencephalography^268^ and head injury especially in those who also reported a loss of consciousness.^269^ It was also reported to have an association with lower olfactory scores in two reports^270,271^ but no association with olfactory identification in a second report.^272^ Flortaucipir binding had no reported association with air particulate matter that had an aerodynamic diameter <10 μm.^273^

In addition to *APOE* ɛ4, bridging integrator 1 rs744373 risk allele carriers showed a higher^274,275^ and faster accumulation of flortaucipir binding, which was exacerbated in the presence of elevated Aβ,^276^ and higher flortaucipir binding was reported to be associated with the PPP2R2B (rs76752255) and IGFBP3 (rs117402302) single-nucleotide polymorphisms,^277^ ‘axon-related’ and lipid metabolism genetic profiles as well as the microtubule-associated protein tau (MAPT) gene.^278^ Flortaucipir binding had no reported association with a total cholesterol polygenic risk score.^279^ In twin pairs, the intensity and extent of flortaucipir binding was reported to be similar suggesting a large role for genetics in the accumulation and spreading of tau.^280^

To fully understand the impact of some of these measures, particularly age, *APOE* ɛ4 and their interaction with Aβ as well as sex, race, socio-economic factors and comorbidities, larger longitudinal studies over longer time periods are required.

## Flortaucipir in atypical Alzheimer’s disease

In addition to typical late-onset amnestic Alzheimer’s disease dementia, flortaucipir has also been used to investigate binding in atypical variants of Alzheimer’s disease, including logopenic variant of primary progressive aphasia (lvPPA), posterior cortical atrophy (PCA) and the behavioural Alzheimer’s disease (bvAD) phenotype.^112,281,282^ In general, these atypical phenotypes tend to occur more frequently in younger Alzheimer’s disease patients, accounting for about a third of patients with early-onset compared with ∼6% of patients with late-onset Alzheimer’s disease ^283^ The topography of flortaucipir binding patterns varies by phenotypes, with a topography consistent within the clinical presentation. Patients with lvPPA may present with asymmetric (L > R) temporo-parietal flortaucipir binding.^14,27,181,190,242,284-288^ PCA may present with greater-than-usual posterior parietal/occipital flortaucipir binding, especially in lateral and inferior aspects.^14,27,140,181,190,242,285-287,289-291^ As for bvAD, flortaucipir binding has been shown to be more heterogeneous but still consistent with the typical Alzheimer’s disease temporoparietal pattern.^14,286,292,293^ In addition, flortaucipir binding patterns in atrophy-defined subtypes of Alzheimer’s disease (e.g. ‘typical’, ‘limbic-predominant’, ‘hippocampal-sparing’, and ‘mild atrophy’) also differ, with the greatest neocortical binding observed in hippocampal sparing and typical patients, while limbic predominant patients showed particularly high entorhinal binding.^176,294,295^ Furthermore, regardless of the clinical phenotype, Alzheimer’s disease patients with younger ages at onset tend to have more intense neocortical flortaucipir binding compared with typical, late-onset Alzheimer’s disease cases.^14,140,184,190,241-245,248^ Across atypical variants, as with typical late-onset amnestic Alzheimer’s disease, elevated flortaucipir binding, global, regional or voxelwise, is associated with cognitive impairment/decline,^16,140,190,242,284,287^ prospective atrophy,^191^ longitudinal florbetapir accumulation,^181,285,296^ neurodegeneration measured with structural MRI,^27,181,184,190,284,287^ synaptic dysfunction measured with FDG-PET,^14,181,184,190,286,290,293^ fractional anisotropy^181^ and aberrant functional connectivity.^297^

Flortaucipir binding patterns in autosomal dominant Alzheimer’s disease (ADAD) have also been investigated.^298-305^ These studies overall showed flortaucipir binding elevation in signature Alzheimer’s disease temporo-parietal regions, with a topography consistent with available evidence in typical, late-onset Alzheimer’s disease. However, ADAD cases tended to show a greater magnitude of flortaucipir binding compared with sporadic typical late-onset Alzheimer’s disease.^301,305^ Again, medial and inferior lateral temporal flortaucipir binding was associated with cognitive impairment.^300,302,303^ Regional flortaucipir binding was elevated in medial and inferior lateral temporal, as well as posterior cingulate regions, in presymptomatic presenilin 1 (PSEN1) E280A ADAD mutation carriers.^298-300^ Of note, in PSEN1 E280A ADAD, early entorhinal flortaucipir binding elevation was associated with striatal amyloid-PET binding.^304^ In Down syndrome (DS), the trisomy 21 causes a duplication of the amyloid precursor protein gene, leading to over-production of amyloid beta.^306^ Flortaucipir studies in DS have reported elevated binding spatially consistent with observed findings in typical Alzheimer’s disease^307-311^ and that neocortical flortaucipir binding is associated with cognitive decline,^309,311^ even when limited to subthreshold amyloid accumulation.^308^

## Flortaucipir in non-Alzheimer’s disease neurodegenerative conditions

While flortaucipir is not intended for diagnostic use in neurodegenerative and neurological conditions other than Alzheimer’s disease dementia, it has been used for research purposes.^308^ Overall, flortaucipir binding elevation has been described in regions in which tau pathology or neurodegeneration would be expected, e.g. basal ganglia in PSP or anterior temporal lobes in semantic variant primary progressive aphasia (svPPA; see below). Several caveats, however, should be considered for a comprehensive interpretation of such findings. First, post-mortem autopsy and tissue studies have repeatedly demonstrated weak/null flortaucipir binding to non-Alzheimer’s disease pathology aggregates, suggesting that *in vivo* findings may be capturing processes that co-localize or associate with the given underlying pathology. Elevated flortaucipir binding in non-Alzheimer’s disease conditions, with some exceptions such as in PART, should thus not be interpreted as evidence for tau pathology. Second, such elevations are usually observed at the group-level, with consistently lower effect sizes compared with findings in Alzheimer’s disease, not being informative or specific at the single-subject level. Third, such elevations, when present, are observed in regions not relevant for Alzheimer’s disease. Similarly, regional flortaucipir PET signal has been shown to accurately discriminate (reaching an AUC of 0.99) between high ADNC and frontotemporal lobar degeneration (FTLD) at autopsy.^41^ Fourth, flortaucipir binding elevation in non-Alzheimer’s disease neurodegenerative conditions in most cases does not track with disease severity, unlike the robust associations observed with clinical and cognitive decline in Alzheimer’s disease.

### Suspected or definite non-Azheimer’s disease tauopathies

One of the most intense areas of flortaucipir research has been non-Alzheimer’s disease tauopathies. Flortaucipir is useful and reliable in detecting tau NFTs in Alzheimer’s disease, thus is thought to be specific for binding to tangles with a paired helical filament (PHF) morphology and a balanced combination of 3R/4R tau isoforms. In non-Alzheimer’s disease tauopathies, tau aggregations may present in different conformations (e.g. straight filaments) and with different isoforms ratios [e.g. 3R-dominant in Pick’s disease or 4R-dominant in progressive supranuclear palsy (PSP) and corticobasal degeneration (CBD), as well as with mixed 3R/4R tauopathy such as in PART or in chronic traumatic encephalopathy (CTE)].^312,313^ Within these conditions, flortaucipir PET-to-autopsy studies have provided contrasting evidence, and some studies noting a correlation between flortaucipir *in vivo* and post-mortem tau neuropathology in patients with CBD,^314^ partially in argyrophilic Grain disease,^315^ but not in PSP,^316^ and with contrasting evidence in CTE.^317^ Of note, *in vitro* studies have more consistently demonstrated weak/null flortaucipir binding to various non-Alzheimer’s disease pathologies.

### 3R- or 4R dominant non-Alzheimer’s disease tauopathies

Overall, *in vivo* topography of flortaucipir binding elevation in PSP has been observed in basal ganglia and subcortical structures across clinical variants,^318-330^ correlating with volume loss, cortical thinning and^331^ gait abnormalities^332^ and associating with neuroinflammation assessed by ^11^C-PK11195-PET.^333,334^ Similar findings have been observed in cortico-basal syndrome (CBS, suspect CBD), with elevation mostly observed in cortico-spinal and motor cortical/frontoparietal areas, as well as in basal ganglia and subcortical structures,^325,330,335-339^ although to a lower magnitude compared with Alzheimer’s disease,^339,340^ or not significantly different from CN.^329^

Recently, a study conducted on a mixed cohort of suspected 4R-dominant tauopathies (i.e. PSP and CBS) described a significant negative correlation between flortaucipir binding and synaptic density with ^11^C-UCB-J-PET.^330^ Similar flortaucipir binding patterns involving subcortical and frontal/temporal regions, including white matter, have been observed in behavioral variant frontotemporal dementia (BvFTD)^315,341,342^ and in non-fluent variant primary progressive aphasia (nfPPA),^288,343^ as well as in nfPPA/apraxia of speech patients with definite Pick’s disease.^203,325,344^ In keeping with cross-sectional evidence, longitudinal studies showed higher rates of change for flortaucipir in PSP, although not correlating with functional decline^345^ and in MAPT Q351R^346^ with an expected topography. Such flortaucipir changes were also observed in patients with apraxia of speech, either primary progressive or in the context of nfPPA,^343,347^ although no difference in binding elevation for PSP versus controls is reported.^348^

### 3R/4r non-Alzheimer’s disease tauopathies

One study showed evidence of elevated flortaucipir binding in medial temporal lobe structures in some suspected non-Alzheimer’s disease pathophysiology cases (SNAP), likely due to PART. A follow-up flortaucipir scan, however, showed that such binding did not increase over time.^349^ Another study found significant and similar associations between medial temporal lobe flortaucipir binding and atrophy, regional thickness and cognition in both amyloid-positive and amyloid-negative (likely PART) groups, suggesting that flortaucipir may be sensitive to tau pathology in PART.^350^ However, other reports suggest that flortaucipir in the medial temporal lobe does not reliably detect NFT in patients with definite PART as secondary pathology at autopsy.^325^ Overall, the available data do not support the use of flortaucipir for the detection of PART, at the same time suggest that the presence of PART is unlikely to have considerable impact on a flortaucipir signal pattern.

Flortaucipir binding has been studied in patients with head trauma, traumatic brain injury (TBI) and repetitive TBI, as well as CTE. In participants with war-related TBI and/or PTSD, mild and diffuse flortaucipir was reported across frontal,^351,352^ parietal^352^ and temporal^351,352^ cortices as well as in the cerebellum.^351^ Additionally, significant correlations were reported between flortaucipir binding and neuropsychological impairment in subjects with PTSD or PTSD + TBI.^352^

In war veterans as well as in individuals with repetitive TBI and suspected or at-risk CTE, patchy, dot-like patterns of flortaucipir binding were reported across the cortex or at the grey matter/white matter junction, a typical observation in CTE pathology post-mortem,^353-355^ as well as in superior frontal, medial temporal and left parietal areas.^355,356^ Participants with TBI and suspected or at-risk CTE had group-level elevated flortaucipir binding in the striatum and substantia nigra^357^; however, overall they exhibited a high degree of heterogeneity at the individual level.^353,354,357^ Additionally, war veterans with elevated flortaucipir binding also presented with elevated plasma NfL levels, an axonal injury marker.^355^

In a study including American football players, flortaucipir binding in cortical regions was found to associate with years of play at the group-level.^356^ Furthermore, flortaucipir binding in similar contact sport populations has been reported to be higher in *APOE* ɛ4 but not in MAPT haplotype H1H1 carriers.^358^ In some subjects with elevated flortaucipir binding and TBI, including sports or accident-related TBI, flortaucipir binding was found to associate with functional connectivity and white matter abnormalities.^359^ In amyloid-positive cases with a history of TBI, flortaucipir binding consistently follows Alzheimer’s disease -like patterns of topography and magnitude.^354,360^ Adding to the complexity of flortaucipir findings in CTE, a recent case-study described a lack of correlation between *in vivo* flortaucipir binding and post-mortem tau pathology in a case with confirmed CTE.^317^

Despite several studies in CTE describing flortaucipir binding elevation at the group-level, effect sizes are usually weak, with non-specific topographies, inconsistent clinical/cognitive correlations and no evidence of correlation with post-mortem tau pathology. In keeping with these limitations, flortaucipir is specifically not indicated for use in CTE in the USA.^308^

### Microtubule-associated protein tau mutation carriers

Several studies have investigated flortaucipir binding in MAPT mutation carriers. Of note, MAPT mutations found outside exon 10, such as MAPT R406W, MAPT V337M and MAPT Q351R, tend to show higher flortaucipir binding, potentially associated with 3R/4R tauopathy.^346,361-364^ Flortaucipir binding showed an expected topography involving frontotemporal and subcortical areas in MAPT mutations known to lead to a dominant 4R tauopathy, such as MAPT 10 + 16, MAPT N279K, MAPT S305N, MAPT P301L and MAPT S305I, but with a much lower magnitude to that seen in 3R:4R tauopathies.^315,363,365,366^ One study including a symptomatic carrier of a MAPT G272V mutation showed extensive cortical flortaucipir binding, with autopsy data showing a combination of 3R tauopathy and encephalitis.^364^ As for pre-symptomatic carriers, there is some indication that focal flortaucipir binding elevation can be observed in medial temporal lobe in MAPT R406W, in frontal areas in MAPT L315R, encompassing insula and frontoparietal regions in MAPT P301.^364^ In the same study, however, no binding elevation was observed for other pre-symptomatic MAPT P301L and MAPT S320F carriers.^364^ Finally, the MAPT rs242557 variant has been associated with increased flortaucipir binding in the hippocampus.^367^

### Suspected or definite TDP-43 proteinopathies

Several independent studies have demonstrated elevated flortaucipir binding in svPPA, typically associated with transactive response DNA binding protein 43 (TDP-43) Type-C pathology,^368^ suggesting flortaucipir may bind to targets or processes associated with pathologies other than Alzheimer’s disease tauopathy. These studies have consistently demonstrated asymmetric elevated binding in anterior temporal lobes, often in temporal poles, consistent with the known atrophy and synaptic dysfunction pattern in svPPA,^288,315,369-373^ and potentially associated with neuroinflammation at translocator protein PET.^342^ In contrast to volume loss, flortaucipir temporal binding in svPPA remained stable over time.^374^ TDP-43 pathology is also observed in patients with a C9ORF72 or progranulin (GRN) gene mutation. Several studies have investigated flortaucipir binding in C9ORF72, overall providing mixed evidence of flortaucipir binding restricted to punctate foci,^315^ frontotemporal,^375^ entorhinal cortex,^376^ mild inferior^372^ or heterogeneous frontotemporal, parietal, occipital and cerebellar regions.^366^ Similar heterogenous findings have been observed in GRN mutation carriers, with reports of elevated frontotemporal and parietal binding,^315^ in contrast to null or very mild inferior temporal/cerebellar binding.^366^

### Suspected or definite synucleinopathies

Several studies have investigated flortaucipir binding in conditions associated with underlying α-synuclein pathology, including Parkinson’s disease, dementia with Lewy bodies and multiple system atrophy (MSA). Several studies have demonstrated a lack of noticeable flortaucipir binding as well as no significant changes over time^377^ in Parkinson’s disease patients and Parkinson’s disease patients with MCI, with exceptions restricted to Parkinson’s disease cases with concomitant Aβ positivity.^328,329,378-382^ Multiple studies have, however, described lower flortaucipir binding in the substantia nigra in Parkinson’s disease patients compared with controls, potentially due to neuromelanin loss in the substantia nigra in Parkinson’s disease patients.^381,383-385^ Several other studies have focused on dementia with Lewy bodies patients, describing null or mild flortaucipir cortical binding elevation compared with C/u, especially in temporoparietal and occipital cortices.^380-382,386-391^ Such flortaucipir binding in dementia with Lewy bodies has been observed to associate with Aβ positivity, with relatively higher flortaucipir binding in dementia with Lewy bodies cases with greater amyloid burden,^380,382,386,390^ cognitive impairment^382,385^ or functional connectivity abnormalities,^392^ but not with synaptic density.^388^ Tau positivity, as measured by flortaucipir or CSF P-tau181, was also found to be associated with lower frequency of Parkinsonism and rapid eye movement (REM) sleep behaviour disorder in dementia with Lewy bodies.^391^ Furthermore, flortaucipir binding in dementia with Lewy bodies was found to generally associate with both CSF t-tau and neuropathological tau (both total tau and regional histology NFTs) at autopsy.^390^ One longitudinal study has additionally demonstrated that flortaucipir binding in dementia with Lewy bodies increases over time, especially in posterior areas, correlating with concomitant neurodegeneration and cognitive worsening.^393^ Limited evidence is available for flortaucipir binding in MSA, with one study describing putaminal binding elevation associated with concomitant dopaminergic degeneration.^394^

### Vascular cognitive impairment and dementia

Few studies have investigated flortaucipir binding patterns in subcortical vascular cognitive impairment (SVCI), SVD or vascular dementia. One study demonstrated that subjects with SVCI, even when amyloid-negative, showed increased flortaucipir binding in inferior temporal areas.^395^ Within subjects with SVCI, higher inferior temporal flortaucipir binding was also found to associate with higher cerebral SVD scores, with medial parietal and temporal flortaucipir binding overall associating with cognitive function. Of note, several of these associations between flortaucipir and cognition were also significant when focusing on the amyloid-negative SVCI patients. Another study reported that prevalence of tau positivity, as measured by flortaucipir with quantitation in Braak III/IV regions, was observed to be lower in Aβ+ SVCI subjects when compared with Aβ+ Alzheimer’s disease subjects (25.9% versus 70%).^396^ Furthermore, SVCI subjects who were both Aβ+ and flortaucipir+ showed steeper cognitive decline, greater global cortical thinning, but not decreased hippocampal volume, compared with Aβ+ and flortaucipir negative subjects.^396^ An independent study found a similar prevalence of flortaucipir positivity in a group of patients with SVD and cerebral amyloid angiopathy (CAA), with 5/15 (33.3%) participants showing elevated flortaucipir binding at a visual read.^397^ Another study showed that subjects with probable CAA and an amnestic neuropsychological profile tended to show higher flortaucipir binding in Alzheimer’s disease signature temporoparietal regions compared with CAA subjects with non-amnestic profiles, although no differences in amyloid-PET were observed.^398^

Flortaucipir literature associated with other neurodegenerative and neurological conditions, including prion diseases, is included in Supplementary material.

### *In vitro* studies

The ability of flortaucipir to bind to targets other than 3R/4R Alzheimer’s disease tau pathology has also been investigated with *in vitro* studies. Within tauopathies, flortaucipir was found to have the highest binding to 3R/4R Alzheimer’s disease-like tau pathology, followed by 3R-dominant tauopathies (e.g. Pick’s disease) and 4R-dominant tauopathies (e.g. PSP, CBD, some FTLD-MAPT) and other mixed 3R/4R tauopathies (e.g. CTE).^399-405^ Several studies have demonstrated no or very minimal flortaucipir binding with TDP-43 pathology^400,401,406^ as well as with α-synuclein pathology.^400,401,406^

## Head-to-head comparisons of flortaucipir with other tau positron emission tomography tracers

### In vitro

Several studies have compared flortaucipir binding with binding of other compounds in tissue specimens. Autoradiography studies showed that in Alzheimer’s disease, flortaucipir mostly binds to lesions of tau aggregated in PHFs, including intra-neuronal and extra-neuronal tangles and dystrophic neurites,^400,401^ replicating Braak staging.^45^ The maturity of tau pathology may also have an impact on the strength of flortaucipir binding.^406^ Flortaucipir did not significantly bind to tau aggregated in 4R-dominant straight filaments,^406,407^ beta-amyloid,^401^ synuclein^406,408^ or TDP-43.^399,401,406^ Some reports have highlighted weak flortaucipir binding to 3R-dominant Pick’s disease^399,400,406^ and FTD with Parkinsonism-17^400^ tau lesions. Very similar properties were observed in head-to-head studies comparing flortaucipir with ^18^F-florquinitau (MK6240),^409^  ^18^F-THK5351,^399,410^  ^18^F-THK5117^410^ and ^11^C-PBB3,^402,410^ with perhaps some evidence for relatively stronger binding of PBB3 in both 4R-dominant and 3R-dominant tauopathies.^402^

### In vivo

A few previous studies have compared flortaucipir binding patterns with the patterns observed for other novel tau-PET radioligands *in vivo* in the same patients, including THK5351,^411^  ^18^F-RO948^412^ and florquinitau (MK6240).^413^ Compared with THK5351, flortaucipir showed correlated, but higher, binding in Alzheimer’s disease patients and lower binding in FTD. The higher binding showed by THK5351 in non-Alzheimer’s disease was thought to reflect other neurodegenerative processes, given that THK5351 strongly binds to monoaminoxidase (MAO) B.^411,414^ Notably, flortaucipir showed less off-target binding in brainstem, white matter and striatum when compared with THK5351. Compared with RO948 in a cohort mainly of Alzheimer’s disease dementia patients, flortaucipir showed lower binding in the entorhinal cortex and higher binding in the striatum, thalamus and choroid plexus, whereas skull/meningeal off-target tracer binding was noticeably higher with RO948.^412^ Of note, neocortical binding was very highly correlated across the neocortex, peaking in the Braak III/IV stage ROI (Spearman rho = 0.96). Both tracers showed increased off-target binding with older age.^412^ When compared with florquinitau in a cohort mostly including cognitively normal controls, the SUVrs generally were highly correlated (*R*^2^ ≥ 0.92), except in the Braak II regions, possibly due to higher adjacent off-target binding evident with flortaucipir. Of note, flortaucipir showed higher striatal/choroid plexus off-target binding, whereas florquinitau showed higher meningeal off-target binding.^413^ Similar relationships between flortaucipir and florquinitau were observed in a study on ADAD.^415^

Although not coming from head-to-head studies, supporting evidence for similar properties across these radioligands is provided by a cross-sectional study pooling flortaucipir, RO948 and florquinitau data.^416^ This study demonstrates that tau-PET binding across several regions, e.g. entorhinal cortex or a temporal composite ROI, provides similar accuracy in differentiating Alzheimer’s disease versus non-Alzheimer’s disease or cognitively normal participants across tracers.^416^

Flortaucipir was used as a comparison standard to evaluate performances of a new putative radioligand, ^18^F-N-methyl-lansoprazole, for which a favourable clinical translation was not exhibited.^417^

## Flortaucipir technical considerations

Flortaucipir-PET scans can be evaluated either with a qualitative visual read, assessing binding elevation topography and magnitude, or with different types of quantitation, providing precise binding estimates.

### Visual read

The FDA-approved method to visually evaluate a flortaucipir scan enables the evaluation of both the presence and extent of elevated binding. This protocol includes reorientation of the image, delineation of a cerebellar ROI and relative windowing of the colour scale to evaluate the presence of binding 1.65 times greater than the cerebellum. When such binding is consistently observed in the posterior lateral temporal/occipital or parietal areas, the image is read as positive, regardless of frontal involvement. This method has been developed to improve the signal-to-background ratio and improve accuracy in detecting flortaucipir signal elevation, demonstrating high sensitivity, specificity and accuracy in predicting Alzheimer’s disease pathology at autopsy.^36^ Based on the localization of the elevated binding, the Alzheimer’s disease pattern can be classified as moderate (elevation limited to lateral posterior temporal/occipital) or advanced (elevation involving parietal regions or lateral posterior temporal/occipital extending to frontal regions).^36,52^ The advanced Alzheimer’s disease pattern is associated with a greater risk of cognitive decline over 18 months in patients with MCI or Alzheimer’s disease dementia.^52^ A recently described visual read protocol for flortaucipir includes both a quantitative score based on binding magnitude (from 0 for no binding to 2 for intense binding) in seven different ROIs and a qualitative pattern evaluation.^418^ The visual scores were associated with flortaucipir cortical SUVrs, clinical diagnosis and amyloid status, with the Alzheimer’s disease patterns also demonstrating efficacy for identifying amyloid-positive MCI and Alzheimer’s disease dementia patients.^418^

### Quantitation

Quantitation approaches are currently the standards of choice in research and clinical trials, both for estimation of individual-level and group-level changes in longitudinal data. Flortaucipir quantitation is generally performed with an ROI-based approach, generating a ratio between average binding in a target region and average binding in a reference region (SUVr). Target regions should be the areas indicative of underlying Alzheimer’s disease pathology (e.g. areas included in the Braak NFT staging^29^), and reference regions should be unaffected by the disease process. Common target regions include areas involved earlier in disease stage, (e.g. entorhinal cortex or inferior temporal areas), as well as larger regions involved in later disease phases [e.g. the temporal meta-ROI^289^ or the multi-block barycentric discriminant analysis ROI].^419^ The most used reference region is the cerebellar grey matter/crus,^420^ although sometimes only inferior and composite white matter areas (PERSI-defined reference^421,422^ or the centrum semiovale^423^) were employed. Different implications for cross-sectional or longitudinal studies should be considered for the different reference regions.^424^ One recent study also evaluated the possibility of a dual reference tissue approach where cerebellar grey matter and subcortical white matter are used to derive different parameters from dynamic flortaucipir scans [relative delivery (R1) and distribution volume ratios (DVRs), respectively].^425^ While the choice of target/reference region is usually largely consistent across groups, there are several sources of variability intrinsically associated with quantitation, which may limit the comparability of findings. These include (but are not limited to): (i) native versus template-space processing; (ii) use of atlas-defined ROIs and the use of different atlases; (iii) MRI-based versus PET-only pipelines^426,427^; (iv) different structural MRI segmentation software (e.g. Statistical Parametric Mapping, FreeSurfer); (v) utilization of partial volume correction approaches and (vi) use of different statistical approaches (e.g. median versus mean). Specifically for longitudinal data and the ability to measure change over time, a recent study comparing >400 combinations of methodological choices indicated that the use of larger temporal meta-ROIs, longitudinal analytical pipelines, composite WM reference regions, two-class partial volume correction and median statistics were the optimal choices.^428^

In addition to SUVr values derived from short scans (e.g. 20 min), quantitation has also been performed on longer dynamic scans to derive other flortaucipir uptake parameters such as binding potentials (BPs) and DVRs. Of note, BP and DVR values have been found to be significantly and tightly associated with SUVr values.^429-431^ Further, several groups have employed voxelwise analyses to investigate flortaucipir binding patterns in Alzheimer’s disease and related conditions, both cross-sectionally and longitudinally, and their relationship with cognitive impairment and decline.^16,63,97,145,178,181,235,432^ More recent approaches under development include whole-brain pattern analysis and machine learning techniques, e.g. Tau^IQ^  ^433^ or convolutional neural network approaches.^434^

### Off-target binding

Flortaucipir-PET studies showed significant off-target binding, hypothesized to be associated with: iron deposits, bleeding, calcifications, neuromelanin, leptomeningeal melanin, MAO A/B or their combination. In flortaucipir human scans, off-target binding is visually evident in the striatum, brainstem and choroid plexus and less frequently in the meninges and dorsal cerebellum (see^435,436^ for reviews). PET/MRI studies revealed a possible association between off-target binding and iron deposits/accumulation.^437,438^ Regarding cerebral bleeding, flortaucipir binding was found to associate with microbleeds,^438^ chronic infarctions^439^ and subacute infarction,^437^ with negative findings in patients with unilateral occlusion of pre-cerebral arteries.^440^ A few studies have reported on possible elevation of flortaucipir binding in meningiomas.^439,441^ Autoradiography studies have showed saturated flortaucipir binding in substantia nigra in melanoma cells^442^; strong off-target binding to neuromelanin and melanin-containing cells, some weak binding to areas of haemorrhage^409^; binding to neuromelanin-containing neurons in substantia nigra,^408^ as well as in leptomeningeal melanocytes adjacent to ventricles and midbrain.^408^ In the same study, binding to choroid plexus was only observed in subjects with leptomeningeal melanocytes within choroidal stroma.^408^ Another study demonstrated that epithelial cells in the choroid plexus actually include tangle-like structures (corresponding morphologically to Biondi ‘ring’ tangles), potentially qualifying flortaucipir binding in the choroid plexus as on-target.^443^ As for MAO, although contrasting evidence has been reported,^409,410,444-448^ recent reports have demonstrated that MAO-A binding is not a significant contributor to the observed PET signal in Alzheimer’s disease and that there is only weak binding of flortaucipir to MAO-B *in vitro* at micromolar concentrations.^448^ As for MAO-A, despite low nanomolar affinity, flortaucipir binding displays fast dissociation rates, leading to a non-significant contribution to the observed PET signal in areas with significant tau pathology burden.^448^ Overall, it is suggested that its contribution to off-target binding for flortaucipir is very weak or minimal,^448^ especially considering the difference in flortaucipir binding patterns in different conditions such as Alzheimer’s disease versus PSP or Parkinson’s disease.

Off-target binding, however, might be problematic in specific circumstances. For instance, off-target signal in the basal ganglia may impact the quantitation of basal ganglia signal elevations in non-Alzheimer’s disease neurodegenerative conditions such as PSP or CBD, which is likely to already be affected by the lower affinity of flortaucipir for non-Alzheimer’s disease tau. Off-target signal in the choroid plexus, on the other hand, might impact quantitation in medial temporal lobe regions aimed at earliest detection of Alzheimer’s disease tau accumulation. Specifically for the hippocampus, several studies have attempted to correct for off-target binding to provide more accurate flortaucipir binding estimates.^138,449,450^ Regardless of localization, the relative impact of off-target binding in imaging studies should also be considered in light of its relationships with age,^451^ sex^452^ and race.^453^

## Summary

A thorough evaluation of the literature pertaining to flortaucipir has been presented. A number of autopsy studies evidence that flortaucipir represents an accurate *in vivo* detection and quantitation of the density and distribution of neocortical NFT accumulation. Flortaucipir binding is elevated in cases of typical Alzheimer’s disease and MCI due to Alzheimer’s disease as well as in individuals who are amyloid positive. In fact, evidence suggests that an elevated flortaucipir scan has a high positive predictive value for an elevated amyloid-PET scan and could be used to indicate the presence of both NFT and Aβ plaque pathology.

In addition to amyloid PET markers, the literature suggests moderate to strong concordance for flortaucipir with both CSF and blood-based biomarkers of Aβ and tau and particularly with phosphorylated tau compounds. Increased density and extent of flortaucipir binding have been shown to correlate with worse deficits on cognitive tests. Additionally, elevated flortaucipir has been shown to be predictive of future cognitive decline as well as clinical progression from CU to MCI or Alzheimer’s disease, suggesting a potential prognostic role for flortaucipir in Alzheimer’s disease clinical evaluations. There are also reports that the region of elevated flortaucipir binding provides further prognostic information regarding the domain of cognitive deficit, with several reports identifying elevated flortaucipir binding in the medial temporal lobe associated with memory loss. Reports suggest that flortaucipir has a stronger correlation with neurodegeneration and cognition compared with amyloid-PET, supporting the amyloid cascade hypothesis where elevated global amyloid precedes elevated tau which in turn precedes neurodegeneration.

There are conflicting or sparse reports on the impact of factors such as *APOE* genotype, sex, race, age, socio-economic factors and comorbidities on the level of flortaucipir binding observed. None of the literature, however, appears to suggest any impact of these factors on the relationship between flortaucipir PET signal and the distribution and density of tau pathology. Increased numbers as well as more longitudinal studies are required to fully understand how these factors and their interplay affect flortaucipir binding. In cases of atypical Alzheimer’s disease, the pattern of flortaucipir signal elevation has overall been reported to be consistent with the expected topography and patterns based on autopsy data. In cases of non-Alzheimer’s disease neurodegenerative conditions, the findings are mixed and depend on the tauopathy or condition under study. The use of flortaucipir is considered to be limited in these studies. In general, flortaucipir appears to bind more consistently, as expected, in 3R/4R tauopathies, still to a lower extent compared with evidence available in Alzheimer’s disease. However, it should be reiterated that flortaucipir is not indicated for non-Alzheimer’s disease conditions. Flortaucipir demonstrates comparable performance in comparison with other tau PET tracers, including the second-generation tracers. All tracers considered had off-target binding, albeit in different regions, that needs to be addressed.

Overall, the findings reported in this comprehensive review of the literature indicate that flortaucipir is an accurate and useful *in vivo* marker of tau pathology that can improve the diagnostic, and potentially prognostic, landscape for Alzheimer’s disease.

## Supplementary Material

fcad305_Supplementary_Data

## Data Availability

Data sharing is not applicable to this article as no new data were created or analysed in this study.
