# Supplementary material for: A review of the flortaucipir literature for positron emission tomography imaging of tau neurofibrillary tangles
Source: Brain Commun. 2023 Nov 16;6(1):fcad305. doi: 10.1093/braincomms/fcad305 (PMC10768888; doi:10.1093/braincomms/fcad305)
Supplement: fcad305_Supplementary_Data [file fcad305_supplementary_data.docx]

A Review of the Flortaucipir Literature for PET Imaging of Tau Neurofibrillary Tangles – Supplemental Material

Samantha C. Burnham, Leonardo Iaccarino, Michael J. Pontecorvo, Adam S. Fleisher, Ming Lu, Emily C. Collins, Michael D. Devous, Sr.

Eli Lilly and Company, Indianapolis, IN, USA

Corresponding Author:

Samantha C. Burnham, Ph.D.

Eli Lilly and Company

3711 Market St.

Philadelphia, PA 19104

781 540 3740

burnham_samantha@lilly.com

## Flortaucipir and non-Amyloid PET tracers

The relationship between neuroinflammation and flortaucipir PET signal has been assessed with the PET tracers ^11^C-PK11195^1;2^ or ^11^C-PBR28.^3^ One group^1^ found no relationship between flortaucipir and ^11^C-PK11195 in Aβ+ AD and MCI subjects whereas another^2^ found that subsequent increases in flortaucipir binding were associated with increases in ^11^C-PK11195 binding. Positive regional correlations between levels of microglial activation, as assessed with ^11^C-PBR28, and tau aggregation, as assessed with flortaucipir, in both MCI (regardless of amyloid status) and AD subjects were found.^3^ Others^4;5^ evaluated the relationship between flortaucipir and synaptic density with the ^11^C-UCB-J PET tracer. Significant negative relationships between flortaucipir and ^11^C-UCB-J suggested that elevated tau pathology is associated with lower synaptic density.

Another previous study used flortaucipir-PET and [^18^F]Fluoro-m-tyrosine ([^18^F]FMT) to investigate relationships between tau burden and catecholamine synthesis capacity in cognitively-normal older adults.^6^ The authors found that, in amyloid-positive subjects, higher synthesis capacity in the locus coeruleus (related to dopamine and norepinephrine) was associated with lower flortaucipir signal and rate of accumulation in a temporal metaROI, suggesting a possible protective effect.^6^

In addition, one study of early frame PET (a surrogate measure of blood flow) demonstrated that hypoperfusion was associated with elevated flortaucipir binding within the amyloid-PET positive group.^7^ Several studies report an inverse relationship between flortaucipir signal and FDG-PET, which measures brain glucose metabolism,^8-12^ that may,^13^ or may not,^8;12^ be dependent on Aβ status and tau resilience.^14^ In keeping with the FDG-PET findings, these data suggest that the presence of NFT pathology, detected by flortaucipir PET, is associated with synaptic dysfunction, brain glucose hypometabolism and hypoperfusion.

Overall, these studies add to the evidence that elevated flortaucipir signal is associated with other measures pathophysiological changes characterizing AD, such as neuroinflammation and vascular changes, as well as with neurodegeneration.

Other neurodegenerative and neurological conditions.

Prion Diseases*.* One study of 4 cases belonging to a familial prion kindred (12-octapeptide repeat insertion in the Prion Protein PRNP gene) found significant neocortical flortaucipir binding elevation, matching AD-signature regions.^15^ Of note, all these cases were symptomatic, presenting with a behavioral-variant FTD syndrome, and were Aβ negative. Another study included two subjects with Gerstmann-Sträussler-Scheinker (GSS) disease, associated with the PRNP F198S mutation, describing elevated flortaucipir binding in frontal, cingulate and insular areas as well as subcortically in the striatum and thalamus.^16^ *In vivo* flortaucipir binding corresponded to localization of pathological tau in 1 subject at autopsy.^16^ However, studies of participants with sporadic Creutzfeldt-Jakob disease found no evidence of increased flortaucipir binding.^17;18^

*Other Conditions.* In addition to the most common proteinopathies and prionopathies described above, flortaucipir binding has also been investigated in other neurological and neurodegenerative conditions. One study found that patients with and without high-convexity tight sulci (HCTS) had a similar likelihood of being Aβ+, but HCTS+ participants specifically showed lower flortaucipir binding and cortical thickness in AD-signature regions compared to HCTS.^19^ One study of Aβ- adult patients with Niemann-Pick type C disease, showed elevated neocortical flortaucipir binding in several patients with a differing topography compared to AD.^20^ One study in Multiple Sclerosis (MS) described a slightly higher frequency of elevated flortaucipir status, although no difference in total cortical flortaucipir binding in MS compared to controls.^21^ Two studies evaluated the potential of flortaucipir in Tuberous Sclerosis Complex (TSC), overall showing multifocal, patchy dot-like patterns of flortaucipir binding mostly in frontotemporal regions, with post-mortem data confirming AD-like tauopathy in these cases.^22;23^ One study of four patients recovering from LGI1 antibody encephalitis, reported elevated flortaucipir binding in two subjects (one with autopsy-confirmed AD pathology) accompanied by hippocampal volume loss.^24^ An a-typical case study of autoimmune encephalitis with leucine-rich glioma-inactivated 1 autoantibodies with AD pathology confirmed at autopsy, demonstrated flortaucipir binding consistent with AD-like topography and magnitude.^25^ Another study did not find flortaucipir binding to be elevated in patients with myotonic dystrophy type 1 (DM1), except in one subject likely with AD comorbidity based on CSF biomarkers.^26^

**References**

1. Parbo P, Ismail R, Sommerauer M*, et al.* Does inflammation precede tau aggregation in early Alzheimer's disease? A PET study. Neurobiol Dis 2018; 117: 211-6.

2. Ismail R, Parbo P, Madsen LS*, et al.* The relationships between neuroinflammation, beta-amyloid and tau deposition in Alzheimer's disease: a longitudinal PET study. J Neuroinflammation 2020; 17(1): 151.

3. Dani M, Wood M, Mizoguchi R*, et al.* Microglial activation correlates in vivo with both tau and amyloid in Alzheimer's disease. Brain 2018; 141(9): 2740-54.

4. Coomans EM, Schoonhoven DN, Tuncel H*, et al.* In vivo tau pathology is associated with synaptic loss and altered synaptic function. Alzheimers Res Ther 2021; 13(1): 35.

5. Mecca AP, Chen MK, O'Dell RS*, et al.* Association of entorhinal cortical tau deposition and hippocampal synaptic density in older individuals with normal cognition and early Alzheimer's disease. Neurobiol Aging 2022; 111: 44-53.

6. Ciampa CJ, Parent JH, Harrison TM*, et al.* Associations among locus coeruleus catecholamines, tau pathology, and memory in aging. Neuropsychopharmacology 2022; 47(5): 1106-13.

7. Raman F, Fang YD, Grandhi S*, et al.* Dynamic Amyloid PET: Relationships to (18)F-Flortaucipir Tau PET Measures. J Nucl Med 2022; 63(2): 287-93.

8. Bischof GN, Jessen F, Fliessbach K*, et al.* Impact of tau and amyloid burden on glucose metabolism in Alzheimer's disease. Ann Clin Transl Neurol 2016; 3(12): 934-9.

9. Scholl M, Lockhart SN, Schonhaut DR*, et al.* PET Imaging of Tau Deposition in the Aging Human Brain. Neuron 2016; 89(5): 971-82.

10. Botha H, Mantyh WG, Murray ME*, et al.* FDG-PET in tau-negative amnestic dementia resembles that of autopsy-proven hippocampal sclerosis. Brain 2018; 141(4): 1201-17.

11. Hanseeuw BJ, Betensky RA, Schultz AP*, et al.* Fluorodeoxyglucose metabolism associated with tau-amyloid interaction predicts memory decline. Ann Neurol 2017; 81(4): 583-96.

12. Sintini I, Schwarz CG, Martin PR*, et al.* Regional multimodal relationships between tau, hypometabolism, atrophy, and fractional anisotropy in atypical Alzheimer's disease. Hum Brain Mapp 2019; 40(5): 1618-31.

13. Adams JN, Lockhart SN, Li L, Jagust WJ. Relationships Between Tau and Glucose Metabolism Reflect Alzheimer’s Disease Pathology in Cognitively Normal Older Adults. Cerebral Cortex 2018; 29(5): 1997-2009.

14. Duong MT, Das SR, Lyu X*, et al.* Dissociation of tau pathology and neuronal hypometabolism within the ATN framework of Alzheimer's disease. Nat Commun 2022; 13(1): 1495.

15. Jones DT, Townley RA, Graff-Radford J*, et al.* Amyloid-and tau-PET imaging in a familial prion kindred. Neurology Genetics 2018; 4(6).

16. Risacher SL, Farlow MR, Bateman DR*, et al.* Detection of tau in Gerstmann-Straussler-Scheinker disease (PRNP F198S) by [(18)F]Flortaucipir PET. Acta Neuropathol Commun 2018; 6(1): 114.

17. Day GS, Gordon BA, Perrin RJ*, et al.* In vivo [(18)F]-AV-1451 tau-PET imaging in sporadic Creutzfeldt-Jakob disease. Neurology 2018; 90(10): e896-e906.

18. Kim HJ, Cho H, Park S*, et al.* THK5351 and flortaucipir PET with pathological correlation in a Creutzfeldt-Jakob disease patient: a case report. BMC Neurol 2019; 19(1): 211.

19. Graff-Radford J, Gunter JL, Jones DT*, et al.* Cerebrospinal fluid dynamics disorders: Relationship to Alzheimer biomarkers and cognition. Neurology 2019; 93(24): e2237-e46.

20. Villemagne VL, Velakoulis D, Dore V*, et al.* Imaging of tau deposits in adults with Niemann-Pick type C disease: a case-control study. Eur J Nucl Med Mol Imaging 2019; 46(5): 1132-8.

21. Zeydan B, Lowe VJ, Reichard RR*, et al.* Imaging Biomarkers of Alzheimer Disease in Multiple Sclerosis. Ann Neurol 2020; 87(4): 556-67.

22. Liu AJ, Staffaroni AM, Rojas-Martinez JC*, et al.* Association of cognitive and behavioral features between adults with tuberous sclerosis and frontotemporal dementia. JAMA neurology 2020; 77(3): 358-66.

23. Liu AJ, Lusk JB, Ervin J*, et al.* Tuberous sclerosis complex is a novel, amyloid-independent tauopathy associated with elevated phosphorylated 3R/4R tau aggregation. Acta Neuropathol Commun 2022; 10(1): 27.

24. Day GS, Gordon BA, McCullough A*, et al.* Flortaucipir (tau) PET in LGI1 antibody encephalitis. Ann Clin Transl Neurol 2021; 8(2): 491-7.

25. Day GS, Gordon BA, Bucelli RC*, et al.* Leveraging molecular biomarkers to make the common diagnosis in the uncommon patient. J Neuroimmunol 2021; 352: 577474.

26. Laforce RJ, Dallaire-Theroux C, Racine AM*, et al.* Tau positron emission tomography, cerebrospinal fluid and plasma biomarkers of neurodegeneration, and neurocognitive testing: an exploratory study of participants with myotonic dystrophy type 1. J Neurol 2022; 269(7): 3579-87.
